# Swedish women with alcohol dependence: survival and cause-specific mortality compared with matched controls

**DOI:** 10.3389/fpubh.2026.1809351

**Published:** 2026-06-17

**Authors:** Rathi Ramji, Hanne Tønnesen, Mette Rasmussen

**Affiliations:** 1 Department of Care Science, Faculty of Health and Society, Malmö University, Malmö, Sweden; 2 Clinical Health Promotion Centre, Addiction Centre Malmö, Region Skåne, Malmö, Sweden; 3 Department of Health Sciences, Lund University, Lund, Sweden; 4 World Health Organisation Collaborating Centre, Clinical Health Promotion Centre, The Parker Institute, Bispebjerg and Frederiksberg Hospital, University of Copenhagen, Copenhagen, Denmark; 5 National Institute of Public Health, University of Southern Denmark, Copenhagen, Denmark

**Keywords:** alcohol dependence, cause-specific death, Kaplan Meier analysis, life-expectancies, mortality

## Abstract

**Background:**

Over the last decades alcohol intake has decreased among the Swedish men but been on the rise among the women, thus leading to an increased risk of alcohol dependence (AD) and related consequences among the Swedish women. The aim of this study was therefore to compare the mortality between women with AD and population-matched controls.

**Method:**

Data were retrieved from the medical records of 2,037 women with AD at the Addiction Centre in Malmö in 1970–2013 and followed up until 2019. They were matched by sex, age, and calendar year with a control group from the general population in Region Skåne. For persons who died during follow-up, death certificates were obtained from the national registers to obtain time and cause of death. The mortality was compared between the groups as observed vs. expected, cumulative survival was assessed by comparing the survival rates of the women with AD to those of the control group. The cumulative survival was calculated as observed compared to expected.

**Results:**

The cumulative survival and standardized life expectancy were significantly reduced for the women with alcohol dependence compared to the matched controls; *T*1/2 = 21 vs. 11, *p* < 0.05 and OR 1.9 CI 1.73–2.02, respectively. More women with alcohol addiction passed away due to accidental and intended causes of death (15.5% vs. 2.8%, *p* < 0.05). Their cause-specific mortality was significantly increased for mental health disorders, respiratory and digestive diseases, but significantly lower for cardiovascular and cancer diseases.

**Conclusion:**

This study showed that the mortality among Swedish women with long term alcohol dependence was significantly increased, thereby suggesting a need for a special focus on this group.

## 1 Introduction

Alcohol drinking is an important modifiable risk factor for the burden of diseases and injuries (1). In Sweden, alcohol consumption has steadily increased since the late 1950s until it stabilized during 2004–2019, with nearly 10% of the population consuming more alcohol than recommended, which corresponds to more than 17 g of pure alcohol per day (2). Swedish men have been consuming more than women, but the difference is reducing as men's intake is decreasing and women's intake is increasing (2, 3), and alcohol dependency is an increasing problem among Swedish women (4). Moreover, an increased consumption of alcohol among women has also been documented globally (1). A recent study from Germany also reported that women aged 65 years and older were more likely than men of the same age, engaged in risk drinking (5). Given their body composition and metabolic functioning patterns, women tend to experience detrimental effects of alcohol such as alcohol-related organ damage to a greater extent compared to men (4, 6). However, women are often said to experience a broad range of barriers for seeking alcohol treatment, such as low perception of a need for treatment, guilt, shame, engagement in multiple substance abuse, economic factors, and fear of loosing responsibility for their child due to child protection services (6).

Given this situation, there seems to be a need for further knowledge regarding women with alcohol dependency. The primary aim of this study was therefore to evaluate the overall standardized mortality rate (SMR) and the cumulated long-term survival, secondly the life expectancy in birth groups and cause-specific mortality among women with alcohol dependency compared to a matched control population of women from the same geographic area. The primary hypotheses were that the SMR and the cumulative long-term-survival were decreased compared to the control group.

## 2 Methods

### 2.1 Study design

This cohort study was based on register-based clinical data on women with alcohol dependency at the Addiction Centre in Malmö, Sweden in 1970–2013 compared to a matched control population from the local background population. This type of data is routinely collected within the Swedish health-care system and stored in national or regional health registers.

### 2.2 Setting and study population

The Addiction Centre in Malmö is one of the largest clinics in the country serving about 1.3 million inhabitants in the Malmö Region in Southern Sweden providing treatment in accordance with the Swedish national guidelines. It has an inpatient and a major outpatient clinic. The first point of contact with patients happened at the acute clinic where they were registered and diagnosed. The routinely collected patient information and diagnosis have been systematically registered in the medical record system. The patients could have been referred to the clinic or showed up on their own initiative. The period of follow-up took place from first contact until January 2019, death, or migration, whatever came first. Patients included in this study were those with alcohol dependence, identified through diagnoses recorded in the Addiction Centre's clinical medical records, based on the ICD classification system (ICD-8 code 303, ICD-9 code 303, and ICD-10 codes F10.1–F10.2).

The study population included adult women with alcohol addiction and a date of the first visit. Patients seeking treatment for drug abuse, lacking diagnosis, being wrongly referred to the Addiction Centre or lacking a Swedish 10 digits personal identification number (PIN) or other important information for linkage to the death certificate register were not included. Further, some patients had to be excluded due to varying recording traditions, lack of clarity in the handwritten journal archives until digitalization in 2005. To reduce possible related bias, the project database was constructed in collaboration with a retired clinical staff member from the Addiction Centre who had first-hand experience working with the patients being included in this study. The control population was obtained from same region by linking to the Swedish Statistics and matched by sex, age, and calendar time. Furthermore, persons' visiting the Addiction Centre were excluded from the control population.

### 2.3 Data collection and variables

**Figure 1:**
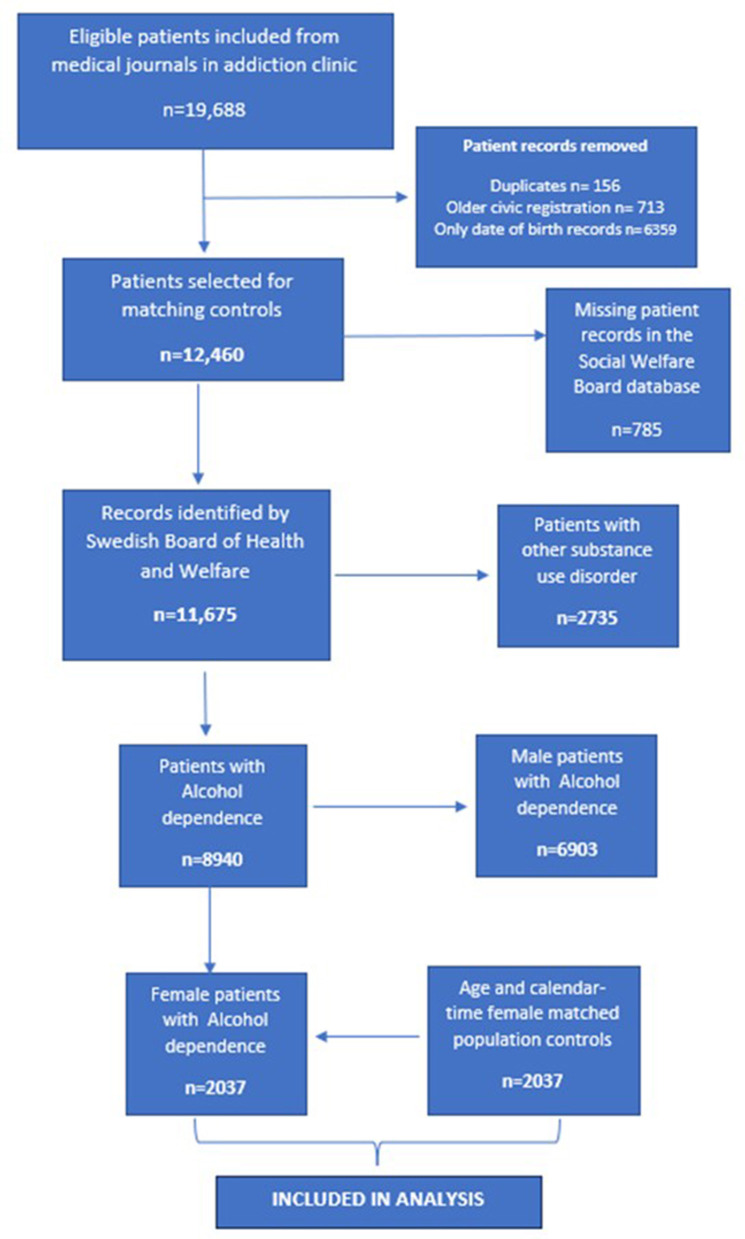
Selection process for patients with alcohol dependence and matched controls.

Data on patients diagnosed with alcohol dependency were collected from the Addiction Centre's medical record systems regarding date of birth and first contact, and sex. A control group was drawn by Statistics Sweden from the general population living in the same region, matched by sex, age, and calendar year. Calendar year here refers to the patients' year of first contact with the Addiction Center, which served as the matching variable to ensure that patients and controls entered the study during the same period. In prior literature, the term “calendar time” has been used to describe matching of controls based on the time when patients enter a study (7). Matching by calendar year ensured that both groups were followed during the same calendar period and were therefore exposed to comparable diagnostic routines, healthcare availability, and societal conditions relevant to that year. Further, data were obtained from the Swedish Causes of Death Register until January 2019, to ensure the variables regarding date of death and causes of death (ICD-codes) for all patients and controls who died during the follow-up period. During the 42-years of the current study the International Classification of Diseases (ICD) changed. Thus, ICD-8 was used in the period 1970–1986, ICD-9 between 1987 and1996, and thereafter ICD-10 (8). To reduce possible bias, the appropriate conversion tools were applied as recommended by the National Board of Health and Welfare to convert diagnoses from ICD-8 and ICD-9 to comparable codes in ICD-10 (8), which was used as the primary diagnostic system. Secondly, the research team collected information related to the cause of death written in the archived medical records if reported, to cross-compare with the information obtained from the national cause of death records. Figure 1 presents the Selection process for patients with alcohol dependence and matched controls.

### 2.4 Data analysis

The SMR was estimated using the ratio of observed to expected mortality rates. The analysis involved indirect standardization for age and calendar time, which were the basis for calculating person-years for the patient group and matched control group, independently. The groups were compared by Chi square test and presented with 95% confidence intervals.

Life expectancy at the age of 20 years was calculated from abridged life tables. At the next step, age-specific mortality rates were converted to the probability of dying, scoring the probability of death as 1 for the oldest age group (≥95 years) over five birth periods: before 1925, 1925–1949, 1950–1974, after 1974. The absolute difference in life expectancy, the 95% confidence intervals, and the gap between the groups were computed. The cumulative survival after the diagnosis of alcohol dependence and the corresponding age among the matched controls was presented as Kaplan-Meier plots and compared by the log rank test. Finally, the groups were compared regarding methods of death (unnatural and natural death) and cause of death by Chi2-test. The data analysis was performed using Stata/IC v.17 (StataCorp).

### 2.5 Ethical considerations

Prior to the initiation of the data assimilation from the registers, the Regional Ethical Committee in Lund approved this study (DNR 2014/506). Although the usual ethical protocol demands the need for informed consent from the concerned patients, it was not possible in the case of the current study since a large proportion of the patient population were deceased or were not in contact with the Addiction Centre when the study was planned. Therefore, the Ethical Committee recommended that the patients should be informed via advertising in the local newspapers with a brief description of the study's name, background, purpose, and implementation. The information was also displayed on Lund University's webpage for a month. The information both in the newspaper and the webpage highlighted that participation was voluntary and if the patient did not wish for their records to be included in the study, they were requested to contact the principal investigator. The research was also registered at the Swedish Authority for Privacy Protection and the GDPR regulations were followed throughout the project. Although the study involved sensitive information related to the patients, the risks were regarded to be outweighed by the importance of the new knowledge to be obtained. This new knowledge will potentially benefit future patients by improving the treatment offered particularly to women diagnosed with alcohol dependency.

## 3 Results

The total cohort of 4074 women; 2037 were patients with a diagnosis of alcohol dependency with a mean age of 45 years (SD = 14.9) and 2037 were age, calendar-time and gender matched controls. Nearly 26% (*N* = 686) of the women patients died during the follow-up period, while 18% (*N* = 358) of the matched controls died during the same period. The standardized mortality rate for women in the patient group was 1.9 (95% CI: 1.7, 2.1), showing that women with alcohol dependency were at a higher risk of dying due to alcohol dependency than controls.

**Figure 2:**
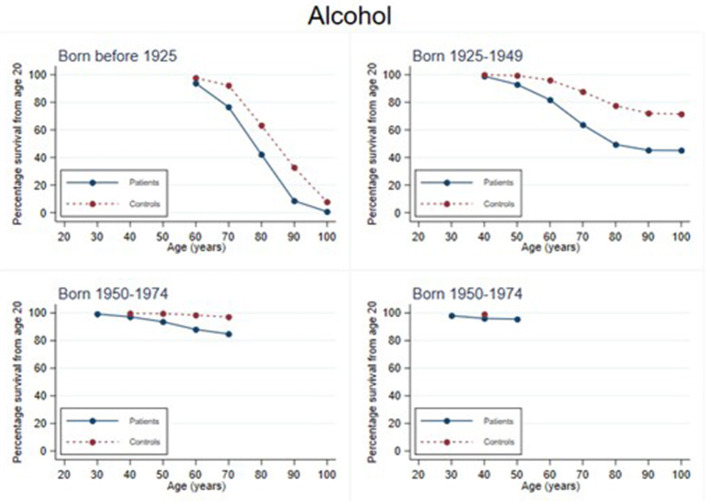
Life expectancy for women patients and matched-controls across birth cohorts.

The life expectancy was significantly reduced for all birth groups, except the youngest women with alcohol addiction compared to their matched controls (Figure 2).

**Figure 3:**
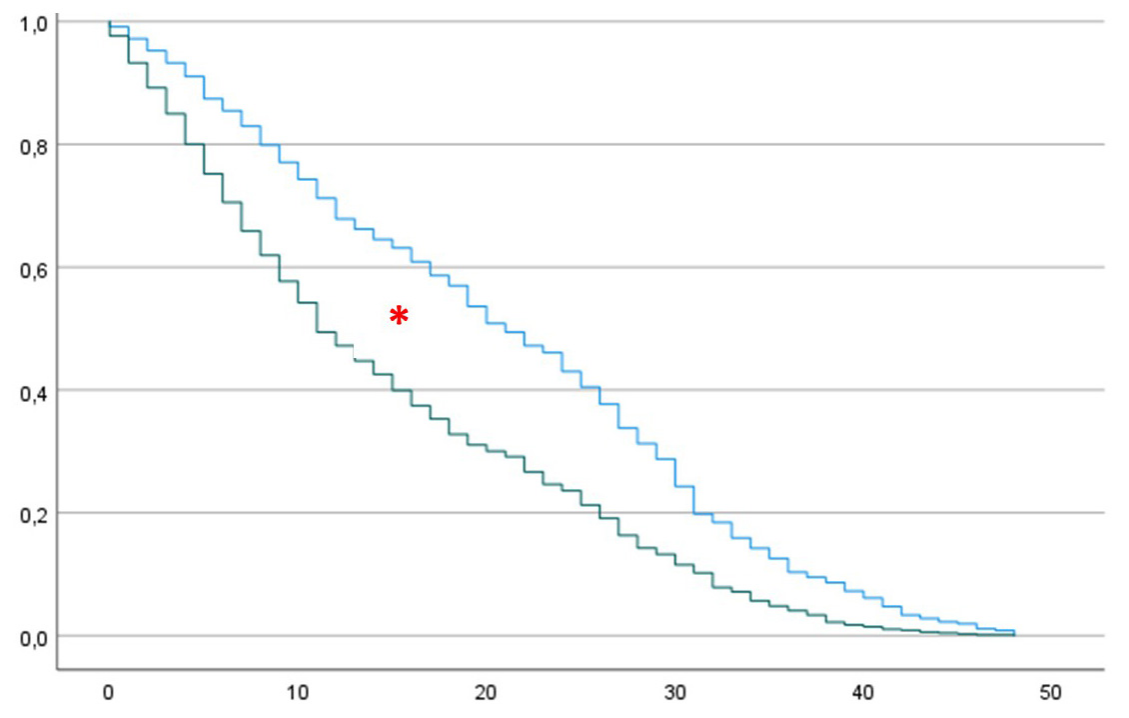
Kaplan–Meier analysis comparing the survival of women patients and matched- controls.

The survival rates from the day of the alcohol dependency diagnosis and a similar day among the control group were also significantly different between the two groups; 14.73 and 20.99 years, respectively; *p* < 0.001 (see Figure 3).

The women with alcohol dependency died more often from unnatural causes of death (15.7% vs. 2.8%, *p* < 0.01) compared to the matched control group. Among the natural causes of death, the mental health disorders (6.4% vs. 2.0%, *p* < 0.01), diseases of respiratory system (13.6% vs. 8.1%, *p* < 0.01), and digestive disorders (6.0% vs. 3.1%, *p* < 0.05) were significantly increased.

In contrast, death from cancer (12.5% vs. 27.1%, *p* < 0.05) and diseases of the circulatory system (23.2% vs. 34.1%, *p* < 0.05) were significantly lower (Table 1).

**Table 1:** Causes of death for women with alcohol dependence and the matched control group.

Main groups	Women (*n* = 686)		Matched controls (*n* = 358)	
	***n***	**%**	***n***	**%**
*Unnatural causes*	^*^108	^*^15.7	10	2.8
*Natural causes:*	578	84.3	348	97.2
Certain infectious and parasitic diseases	13	1.9	7	2.0
Cancer^1^	^*^86	^*^12.5	97	27.1
Endocrine, nutritional, and metabolic diseases	5	0.7	3	0.8
Mental and behavioral disorders	^*^44	^*^6.4	7	2.0
Diseases of the nervous system	20	2.9	7	2.0
Diseases of the circulatory system	^*^159	^*^23.2	122	34.1
Diseases of the respiratory system	^*^93	^*^13.6	29	8.1
Diseases of the digestive system	^*^41	^*^6.0	11	3.1
Diseases of the genitourinary system	5	0.7	4	1.1
Symptoms, signs, and abnormal clinical and laboratory findings, not elsewhere classified	84	12.2	45	12.6
Unknown	30	4.4	16	4.5

^1^Except skin neoplasm: C-49 and cancer in situ: D-00 to D-48.

^*^p-value <0.05.

## 4 Discussion

The results of the current study show that significantly more women with alcohol dependence from all birth groups except the youngest have died in the study period compared to the matched controls. In addition, their cumulative survival after being diagnosed with alcohol dependency was significantly shorter. More have died from unnatural causes, from mental, digestive, and respiratory diseases, but fewer from circulatory and cancer diseases. Malmö is representative for Sweden regarding alcohol intake (Skåne 15.0% risk drinking; The general Swedish population 15.5%; i.e., same prevalence. Skåne women 11,3%—men 17.1%; The general Swedish population 12.5%—men 18.5%; i.e., same difference between men and women = about 6%) (9).

Overall, alcohol-related mortality seems to reduce the life expectancy by about 5 years for women over 45 years of age, despite a general increase in life expectancy in the Swedish population (3, 4). Several other studies have shown an increased mortality caused by alcohol use and decreased life expectancy in women compared to the control populations; amongst others, Swedish studies with a shorter follow-up (10, 11), Baltic and Nordic studies of women diagnosed with alcohol use disorder between 1987 and 2006 (12–14). Other studies in Scandinavia and elsewhere have shown similar results of a higher mortality (15, 16). Similar to a study on women with alcohol use disorder from the US (16), our group of women with alcohol dependence most often died due to mental health disorders and liver disease.

An excessive amount of evidence exists supporting a strong association between negative effects of mental health problems such as trauma, adversity, and chronic stress which in turn would increase the vulnerability induced by alcohol particularly in women compared to men (18, 19). Negative psychological effects such as anxiety, fear, anger, irritability, and sadness are usually heightened in women compared to men and lead to higher emotional dysregulation that lead them toward substance use disorders, which in turn have fatal consequences such as self-injuries, suicide, accidents, violence and other intentional causes of death (19–21). This is also well-aligned with the results of the current study which shows that death due to external causes was higher among the women patients compared to the matched controls.

Although cardiovascular disease happens to be the leading cause of death among women patients, the proportions were lower than that of their matched controls and even that of the men patients. This is probably due to the generally low atherosclerosis associated with a high alcohol intake (21–23). The high frequency of respiratory diseases among both men and women with alcohol dependency may be caused by coexisting smoking, as smoking is much higher in this group among patients with alcohol dependency compared to the background population; in Sweden about 70% and 30% (24).

The lower cancer prevalence in our cohort was surprising, as the literature reports it to be increased in patients with alcohol dependence (1, 25, 26). This is also the case for other Swedish studies like the Stockholm study (11), where nearly 27% of the women with AUD died due to cancer (17, 27). You could consider if a lower screening rate and poorer access and/or use of the healthcare services may cause an underdiagnosing of cancer thus more seldom being added to the death certificates. However, other groups with low screening, diagnosing and treatment of cancer have increased cancer mortality, e.g., patients with severe mental illness (28). In contrast, the lower cancer mortality observed among patients in our cohort may partly reflect early mortality from other causes before cancer has time to develop (17). Another contributing factor could be the low smoking prevalence among women in Sweden, which is known to reduce overall cancer mortality risk (29). Finally, previous research suggests that the stigma associated with alcohol use disorders may discourage individuals from seeking or engaging with healthcare services, potentially leading to underdiagnosis or delayed detection of comorbid conditions such as cancer (30).

Despite the lower cancer prevalence, the results support the need for a paradigm shift toward including women in the target group for prioritized alcohol dependence interventions, even better if combined with preventive activities to postpone the drinking debut and to lower the alcohol intake resulting in fewer individuals with alcohol dependence in the future.

## 5 Strengths and limitations

One of the most prominent strengths of the current study was the availability and accessibility of patient records in the Addiction Centre archives, and in the Swedish Registries such as Statistics Sweden and the Swedish Board of Health and Welfare where medical and demographic data are meticulously recorded. Another strength of this study was that there were a few or almost no changes to the treatment for alcohol dependence offered in the Addiction Centre from throughout the 40-year period. Further, this study included a large sample of women patients followed up for a very long time. Despite these strengths this study did have several limitations. The patient data assimilated may have been biased by errors in manually registered identification details such as id-number and name at each visit. One possible reason for this was disclosure stigma among patients as they often tried to conceal their identities through offering mismatched information when treatment continues over several visits. In addition, some patients arrived at the Addiction Centre under severe influence of alcohol, and they may not have been able to provide the right information about themselves. Thus, the initial data set assimilated from the Addiction Centre had several duplicate records with either matching personal identification number and names. The research team in this study examined the data thoroughly ahead of analysis for possible data gaps together with Addiction Centre staff who could identify and distinguish patients based on the information in their medical records. Duplicate data were identified and rectified for over a 100 patients. Another possible limitation was the inability to include persons who had a personal identification number of nine-digits or less since their death certificates were not available with the Swedish authorities. It must be noted that most of these patients were those born before the year 1965.

Further to development of alcohol dependency, alcohol drinking is often linked to other risky lifestyle behaviors including smoking, malnutrition, and physical inactivity, which also aggravates a broad range of diseases (17). Especially, smoking seems to have confounded the results by smoking-related causes of death like respiratory diseases. These data were seldom included in the medical records and therefore control for the related confounding was not possible (31, 32). A further limitation was that Cox regression and hazard ratios could not be reported, as the medical records did not contain reliable information on patients' duration in treatment or continuous time under observation. Consequently, the analysis relied on standardized mortality ratios and Kaplan–Meier plots, both of which are established and appropriate methods for evaluating long term mortality patterns in cohort studies (33). Furthermore, the generalisability of the results is limited given that the results may not be directly applicable to other contexts aside from the Nordic countries, since other countries in the world have widely contrasting circumstances in relation to alcohol dependence treatment access, routines, alcohol cultures and policies (34).

## 6 Perspectives

### 6.1 Clinical implications

As alcohol consumption increases among Swedish women, it is essential for healthcare, social, educational, and other sectors to intensify their efforts to address this development. This can be achieved by enhancing health literacy regarding alcohol drinking and dependence, without stigmatizing women who need treatment. It is important that clinical staff across all specialities possess the necessary skills for early identification, motivational support, empowerment, and initiation of intervention, as well as referral for treatment of women with alcohol dependence.

### 6.2 Scientific implications

This study assesses the mortality of all women at one of Sweden's largest addiction centres for a period of four decades. Due to the increase in alcohol intake in Sweden among women, it is important to carefully evaluate future interventions. The low cancer-related causes of death should be explored among the men with alcohol dependency at the same addiction centre and other groups.

## 7 Conclusion

This study elucidated that mortality among women with alcohol dependence was significantly higher compared to the matched controls from the background population. While survival was significantly better for women compared to men with alcohol dependence, this was not the case for persons born after 1975 onwards. As the alcohol intake is still increasing among women, there is an urgent need for a targeted focus to address this trend and reduce the frequency and consequences of alcohol dependence among women.

## Data Availability

The raw data supporting the conclusions of this article will be made available by the authors, without undue reservation.
